# Deep attenuation transducer to measure liver stiffness in obese patients with liver disease

**DOI:** 10.1007/s10396-022-01270-y

**Published:** 2022-12-16

**Authors:** Masashi Hirooka, Yohei Koizumi, Yoshiko Nakamura, Ryo Yano, Kana Hirooka, Makoto Morita, Yusuke Imai, Yoshio Tokumoto, Masanori Abe, Yoichi Hiasa

**Affiliations:** 1 Department of Gastroenterology and Metabology, Ehime University Graduate School of Medicine, Shitsukawa 454, Toon, Ehime 791-0295 Japan; 2 Department of Gastroenterology, National Hospital Organization Ehime Medical Center, Yokogawara 366, Toon, Ehime 791-0281, Japan

**Keywords:** Deep attenuation transducer, Liver stiffness, Chronic liver disease, Skin-to-liver capsular distance, c-Convex

## Abstract

**Purpose:**

Deep attenuation transducers (DAX) are capable of imaging at diagnostic depths of up to 40 cm. The feasibility of DAX for liver stiffness measurement (LSM) has not been reported clinically. We aimed to assess the feasibility and reliability of DAX for LSM.

**Methods:**

Overall, 219 patients with chronic liver disease were enrolled. The success rate (acquired after ≥ 10 valid measurements) and inadequate measurements (interquartile range/median ≥ 0.3) for DAX were compared with those of conventional convex (c-convex) probes and M and XL probes of vibration-controlled transient elastography.

**Results:**

LSM was successfully performed for all patients using DAX through all degrees of skin-to-liver capsular distance (SCD). Especially in patients with an SCD ≥ 30 mm, the difference in the rate of acquisition of 10 valid measurements was remarkable: M probe (8/33, 24.2%), XL probe (26/33, 78.8%), c-convex probe (33/43, 76.7%), and DAX (44/44, 100%). In patients with an SCD ≥ 30 mm, the inadequate measurement rate of M probe (1/8, 12.5%), XL probe (8/26, 30.8%), and c-convex probe (6/33, 18.2%) was higher than that of DAX (1/43, 2.3%). The areas under the curve for diagnosis of F4 with shear wave speed by c-convex and DAX were 0.916 and 0.918, respectively. Between DAX and c-convex probes, the intraclass correlation coefficient of 0.937 (95% CI 0.918–0.952) was excellent. Bland–Altman plots revealed that there was no statistically significant bias.

**Conclusion:**

Liver stiffness measured by DAX is feasible and reliable for all patient populations, while the XL probe is limited to use in obese patients.

**Supplementary Information:**

The online version contains supplementary material available at 10.1007/s10396-022-01270-y.

## Introduction

Chronic liver diseases are a major cause of morbidity and mortality worldwide [1, 2], and can lead to liver fibrosis and subsequent portal hypertension, end-stage liver disease, and hepatocellular carcinoma [3, 4]. There is evidence that, when the underlying cause is prevented, liver fibrosis may regress or stabilize [5, 6]. Accurate staging of liver fibrosis may be beneficial in monitoring treatment efficacy and disease progression, and in establishing a prognosis [7, 8]. Recently, to diagnose hepatic fibrosis stage or degree of portal hypertension, liver stiffness measurement (LSM) has been broadly performed [9, 10]. LSM with vibration-controlled transient elastography (VCTE) has been widely evaluated in patients with chronic liver disease.

Obesity is a rapidly growing public health concern, with the worldwide prevalence increasing considerably in the last four decades [11]. Obesity, defined as a body mass index of 30 kg/m^2^, is the strongest predictor of failed (i.e., no valid measurement) or unreliable LSM [12–14]. To overcome this limitation, an XL probe has been utilized for specific use in patients with obesity; however, there are some concerns about the XL probe. Generally, M or XL probes are selected according to subcutaneous fat thickness, but there are no clear criteria for the degree thereof. Furthermore, M and XL probes yield different stiffness values if measured at the same location. Although VCTE is an easy and common method to assess liver stiffness, it does not provide B-mode imaging. Subsequent to the widespread use of VCTE, B-mode-based elastography has also been used extensively [15–17], but it does not offer an XL probe for obese patients. A deep attenuation transducer (DAX) was developed for such patients and has the capability to acquire images at diagnostic depths of up to 40 cm. To the best of our knowledge, the feasibility of DAX used for LSM or splenic stiffness measurement has not been reported clinically. We aimed to assess the feasibility and reliability of DAX for LSM.

## Materials and methods

### Study population

**Fig. 1:**
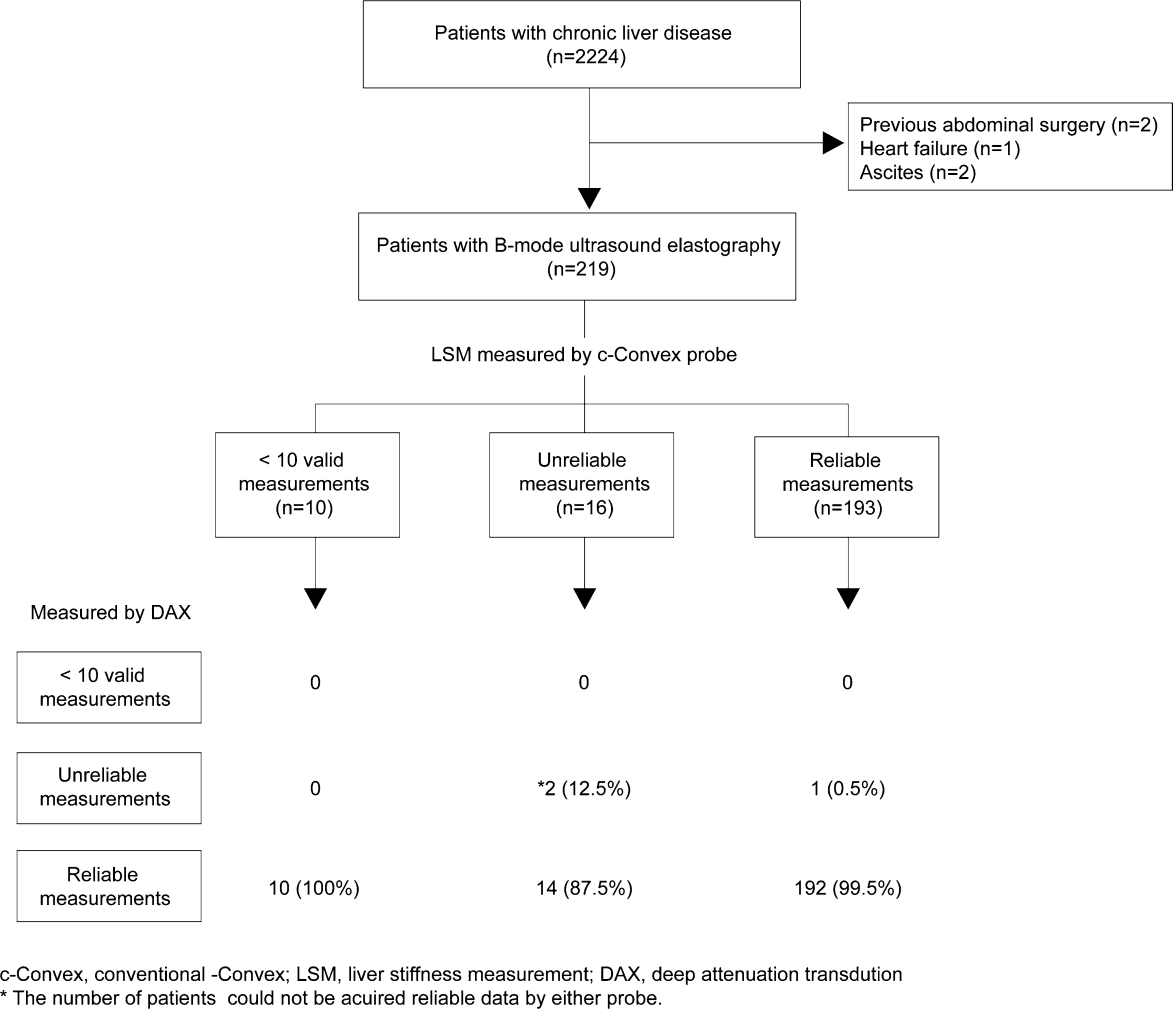
Study design. *c-convex* conventional convex, *LSM* liver stiffness measurement, *DAX* deep attenuation transducer**.** *The number of patients for whom reliable data could not be acquired by either probe

This retrospective study was approved by the institutional ethics committee and was conducted in accordance with the principles of the Declaration of Helsinki. We included patients with chronic liver disease who underwent both VCTE and point shear wave elastography (p-SWE) between July 2018 and April 2022. The inclusion criteria were age 20–90 years, Eastern Cooperative Oncology Group performance status of 0–2, and the ability to follow instructions for breathing control. Notably, sex was not considered during the selection process. Patients meeting any of the following criteria were ineligible: (1) contraindications to LSM (e.g., pregnancy, implantable cardiac devices), (2) previous abdominal operation, (3) accumulation of ascites, and (4) history of heart failure. In total, 219 patients were enrolled (Fig. 1). Of these, liver biopsy was performed for 92 patients within one month.

### LSM using p-SWE

**Fig. 2:**
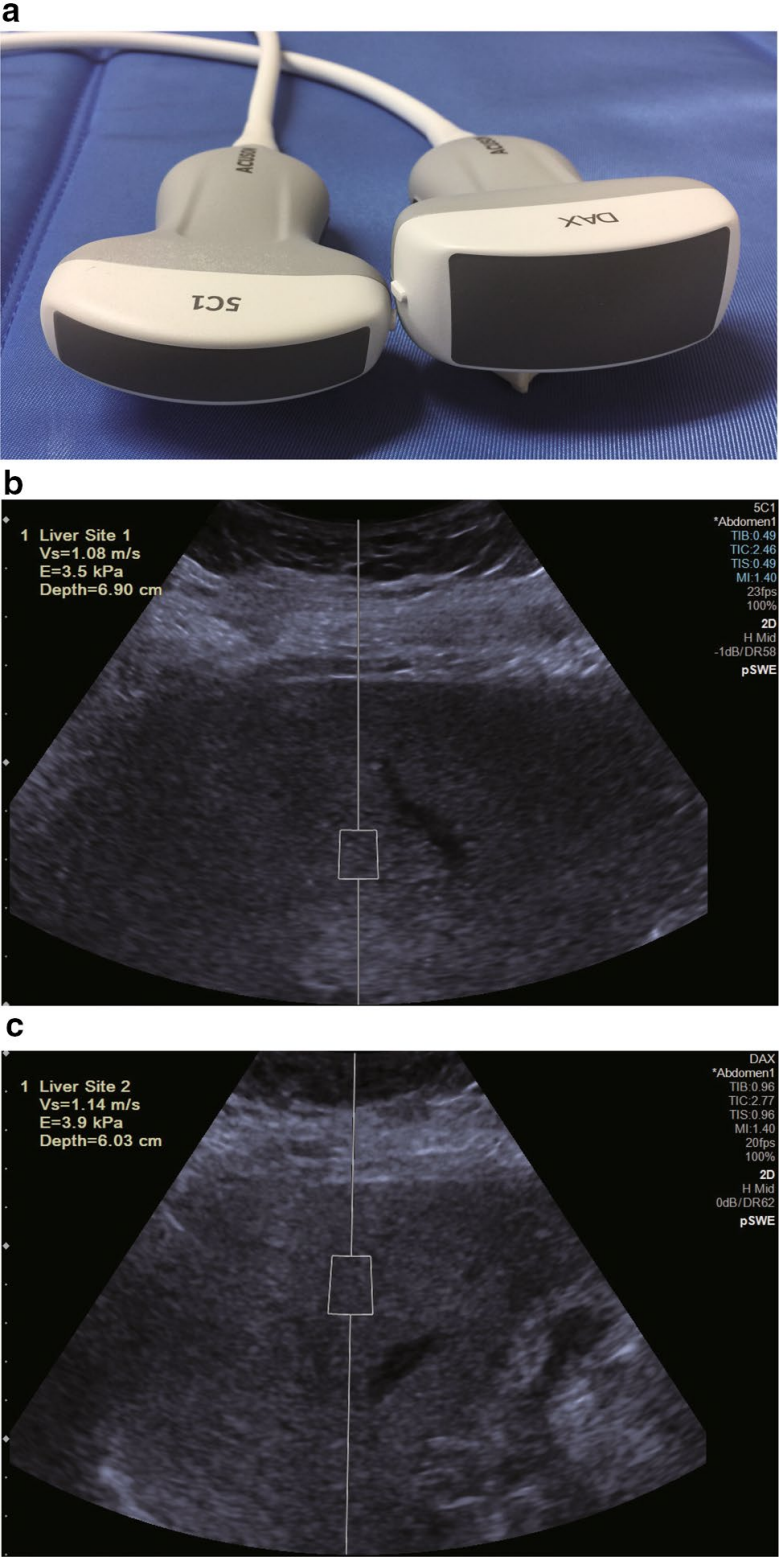
Liver stiffness measurement using DAX and convex probes. **a** c-Convex (left) and DAX (right) probes. **b** Liver stiffness measurement using c-convex probe for patient with SCD of 30 mm. **c** Liver stiffness measurement using DAX for the same patient. *c-convex* conventional convex, *DAX* deep attenuation transducer, *SCD* skin-to-liver capsular distance

P-SWE measurements were performed using the ACUSON Sequoia (Siemens Healthcare K.K., Tokyo, Japan) equipped with a 5C-1 curved transducer and DAX (Fig. 2a) by two operators with > 10 years of experience in LSM. Each patient was placed in the supine position and underwent p-SWE on B-mode imaging after an overnight fast while refraining from smoking cigarettes.

A region of interest (fixed-dimension 1 × 0.5-cm box) in the liver parenchyma, free of large blood vessels and gallbladder, was selected. The detection site was fixed at 1.0–2.0 cm beneath the right liver capsule, using the intercostal approach. The acoustic radiation force impulse (push pulse, m/s) [18] was maintained perpendicular to the liver capsule (Fig. 2b, c). Based on a previous report [18], more than 10 successful measurements were performed for each patient. Thus, the criteria for feasibility were acquired from more than 10 validated measurements, and both the interquartile range (IQR)/median LSM and more than 10 validated measurements < 0.30 were defined as reliable results.

Liver stiffness was measured using VCTE with both M and XL probes from the FibroScan 430 (EchoSens, Paris, France), by the same operators, as previously reported [18]. The criteria for feasibility and reliability remained the same. LSM was performed with and without obesity using the M probe, followed by the XL probe.

### Histological diagnosis

A liver biopsy was performed within 1 week of hospitalization using a cutting needle 1.6 mm in diameter and 15 mm in length. Failure to obtain adequate biopsy specimens (fragment length ≥ 1.5 cm with > 6 portal tracts) led to the exclusion of patients from this study. All liver biopsy samples were fixed in formalin and embedded in paraffin, and sections (4-μm-thick) were stained with hematoxylin–eosin and impregnated with silver. Liver steatosis was scored as follows: S0, < 5%; S1, 5–33%; S2, 33–66%; and S3, > 66%. The presence of inflammation or ballooning was also recorded.

### Statistical analysis

Continuous variables are expressed as the median (IQR) or mean ± standard deviation. The relationship between DAX and the conventional convex (c-convex) probe (5C-1) was determined through Pearson’s correlation coefficient (*r*), which was classified as minimal (|*r*|< 0.2), weak (|*r*|= 0.2–0.4), moderate (|*r*|= 0.4–0.7), or strong (|*r*|= 0.7). Pearson’s correlation coefficient was used to analyze the following correlations: DAX vs. c-convex, M vs. XL probe, and DAX vs. XL probe. The dots that existed outside the area of 95% CI were defined as divergent cases. Bias, defined as the average difference between DAX and c-convex measurements, was assessed using Bland–Altman analysis [19, 20]. The 95% limits of agreement were calculated and shown in Bland–Altman plots. The correlations between the difference and mean of the measurements, fixed error, and proportional error were also calculated. When an inter- or intra-observer correlation was analyzed, Bland–Altman analysis was utilized. Additionally, linearity was evaluated by intraclass correlation coefficients (ICC). Based on the 95% CI of the ICC estimate, values < 0.5, 0.5–0.75, 0.75–0.9, and > 0.90 are indicative of poor, moderate, good, and excellent reliability, respectively [21]. To compare the diagnostic ability for liver fibrosis between DAX and c-convex probe, the areas under the curve (AUCs) determined by receiver-operating characteristic curve analysis were compared using DeLong’s test. To predict the contributing factors for discrepant cases between LSM performed using DAX and c-convex probe, both univariate and multivariate logistic regression analyses were performed. The Kruskal–Wallis test was used for non-parametric data. Multiple comparisons were performed using the Steel–Dwass test. To assess the accuracy of the LSM for differentiating fibrosis stage ≥ 3, we constructed receiver-operating characteristic curves by plotting sensitivity against (1–specificity) at each value. The diagnostic performance of the LSM was assessed by receiver-operating characteristic curve analysis and the index of accuracy was the AUC, with values close to 1.0 indicating high diagnostic accuracy. Most of the statistical analyses were performed using STATA version 15 (StataCorp, College Station, TX, USA). The proportional or fixed error in Bland–Altman analysis was calculated using modified R commander (https://personal.hs.hirosaki-u.ac.jp/pteiki/research/stat/R/). Statistical significance was set at *p* < 0.05.

## Results

### Baseline characteristics

In total, 219 patients (111 women; mean age, 60.8 years) were included in the study. The characteristics of the enrolled patients are shown in Table 1. The median body mass index was 25.4 kg/m^2^. The median skin-to-capsular distance (SCD) was 21.3 mm. Although 13 patients had ascites, stiffness measurements could still be performed.

**Table 1:** Patient characteristics

Factors	
Age (years)	60.8 ± 14.2
Male	109 (49.5%)
Body mass index (kg/m^2^)	25.4 (22.1–30.2)
< 25: ≥ 25, < 30: ≥ 30, < 35: ≥ 35, < 40: ≥ 40	106: 57: 30: 26
Etiology (viral: non-viral)	48: 172
Ascites	13 (5.9%)
Fibrosis stage (F0:F1:F2:F3:F4)	14: 17: 5: 11: 45
Alanine aminotransferase (IU/L)	37 (22–68)
Platelet (× 10^3^/μL)	191.9 ± 88.0
Serum albumin (g/dL)	4.0 (3.5–4.3)
Total bilirubin (mg/dL)	0.8 (0.6–1.2)
Prothrombin time (%)	90.5 ± 20.6
Fasting plasma glucose (mg/dL)	108 (93–136)
Total cholesterol (mg/dL)	182.2 ± 42.7
Triglyceride (mg/dL)	98 (67–136)
High-density lipoprotein cholesterol (mg/dL)	46 (37–59)
Low-density lipoprotein cholesterol (mg/dL)	101 (77–121)
Mac2 binding protein (ng/mL)	1.3 (0.7–3.5)
Skin-to-liver capsule distance (mm)	21.3 (18.0–28.0)
< 20 mm: 20–30 mm: > 30 mm	84: 92: 43

Continuous variables are expressed as median (interquartile range [IQR]) or mean ± standard deviation (SD)

### Inter- and intra-operator reproducibility

**Fig. 3:**
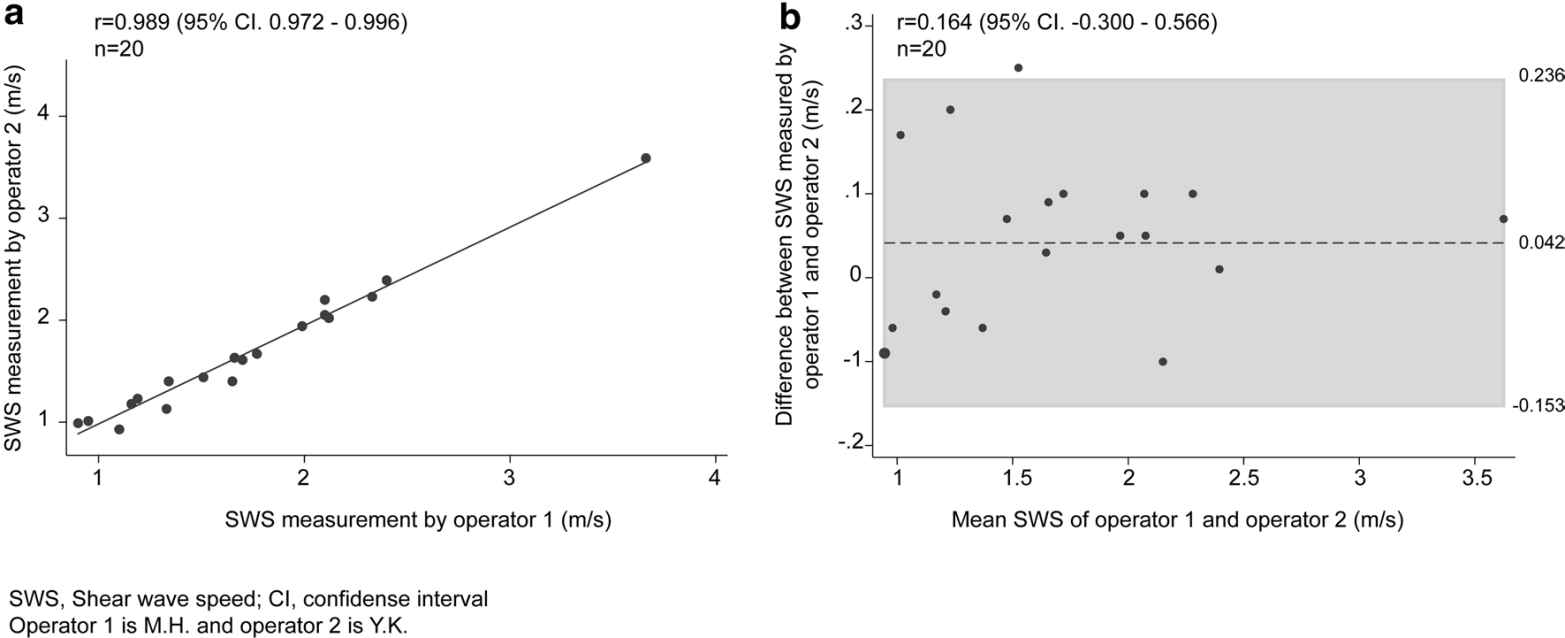
Reproducibility between two operators. **a** Liver stiffness measurements performed by Operator 1 were very well correlated with measurements performed by Operator 2. **b** Bland–Altman plots do not show fixed and proportional bias. *SWS* shear wave speed, *CI* confidence interval

When the two operators (M.H., Y.K.) measured LSM using the DAX probe for the same 20 patients, the relationship between the measurements by the two operators was considered very strong (*r* = 0.989; 95% CI 0.972–0.996), and inter-operator reliability revealed an excellent ICC of 0.987 (95% CI 0.966–0.995) (Fig. 3a). Moreover, to confirm inter-operator reliability, the Bland–Altman method was used; Fig. 3b illustrates the LSM bias between the two operators. Bland–Altman plots revealed there was no statistically significant bias, in which the bias was 0.042 (95% CI − 0.153 to 0.236) and *r* = 0.164 (95% CI − 0.300 to 0.566). There were no significant fixed (*p* = 0.08) and proportional errors (*p* = 0.49). In addition, one operator (M.H.) measured the LSM using the DAX probe for the other 20 patients to confirm the intra-operator reliability. Moreover, regarding intra-operator reliability, an excellent ICC of 0.953 (95% CI 0.882–0.982) was revealed. Bland–Altman analysis revealed that there were no significant fixed (*p* = 0.30) and proportional errors (*p* = 0.21).

### Reproducibility between c-convex probe and DAX

**Fig. 4:**
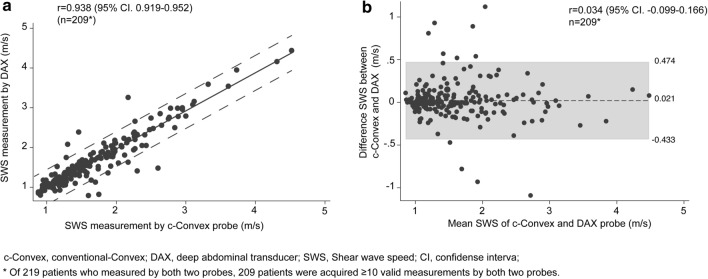
Reliability between liver stiffness measurements performed using different probes. **a** Scatter plots between liver stiffness measurements performed using DAX and c-convex probes. The correlation coefficient was very good. **b** Bland–Altman plots show no fixed and proportional bias. *c-convex* conventional convex, *DAX* deep attenuation transducer, *SWS* shear wave speed, *CI* confidence interval**.** *Of the 219 patients who underwent liver stiffness measurement using both the probes, 209 patients had ≥ 10 valid measurements with both probes

The correlation coefficient of the LSM conventional probe and DAX was 0.938 (95% CI 0.919–0.952) (Fig. 4a). When the operator performed LSM using both the c-convex probe and DAX, different-probe reliability also revealed an excellent ICC of 0.937 (95% CI 0.918–0.952). Bland–Altman plots revealed there was no statistically significant bias, i.e., 0.021 (95% CI − 0.433 to 0.474) and r = 0.034 (95% CI − 0.099 to 0.166) (Fig. 4b). There were no significant fixed (*p* = 0.21) and proportional errors (*p* = 0.38). Univariate and multivariate analysis of clinical parameters to predict divergence of the LSM between the c-convex probe and DAX revealed that only SCD was a significant factor in both analyses (Table 2). As shown in Fig. 4a, six cases were underestimated by DAX, while three cases were overestimated by DAX. The median Mac-2-binding protein glycan isomer (M2BPGi) cutoff index (COI) in patients with an overestimate was superior to that in those with an underestimate (6.8 vs. 1.1, respectively). In only one case (body mass index: 13.8 kg/m^2^), M2BPGi was not consistent with DAX, although M2BPGi was consistent with c-convex.

**Table 2:** Assessment of divergent cases between measurements with c-convex probe and DAX in LSM

Liver				
	Univariate analysis		Multivariate analysis	
	Odds ratio	*p* value	Odds ratio	*p* value
Age > 65 years	0.44 (0.08–2.29)	0.305	0.57 (0.09–3.62)	0.561
Male	0.76 (0.17–3.46)	0.719	0.70 (0.12–3.95)	0.685
BMI > 30	4.13 (0.89–19.05)	0.071	1.02 (0.13–8.36)	0.981
Viral hepatitis	1.45 (0.27–7.73)	0.670	4.16 (0.46–37.06)	0.204
SCD > 30 mm	5.77 (1.24–26.79)	0.025	18.08 (1.26–258.87)	0.033
Ascites	96,563.12 (0–NE)	0.993	42,356.74 (0–NE)	0.997
M2BP	0.91 (0.79–1.07)	0.316	0.83 (0.69–1.06)	0.330

*BMI* body mass index, *SCD* skin-to-liver capsule distance, *M2BP* Mac2 binding protein

### Successful and adequate LSMs for each SCD category

**Fig. 5:**
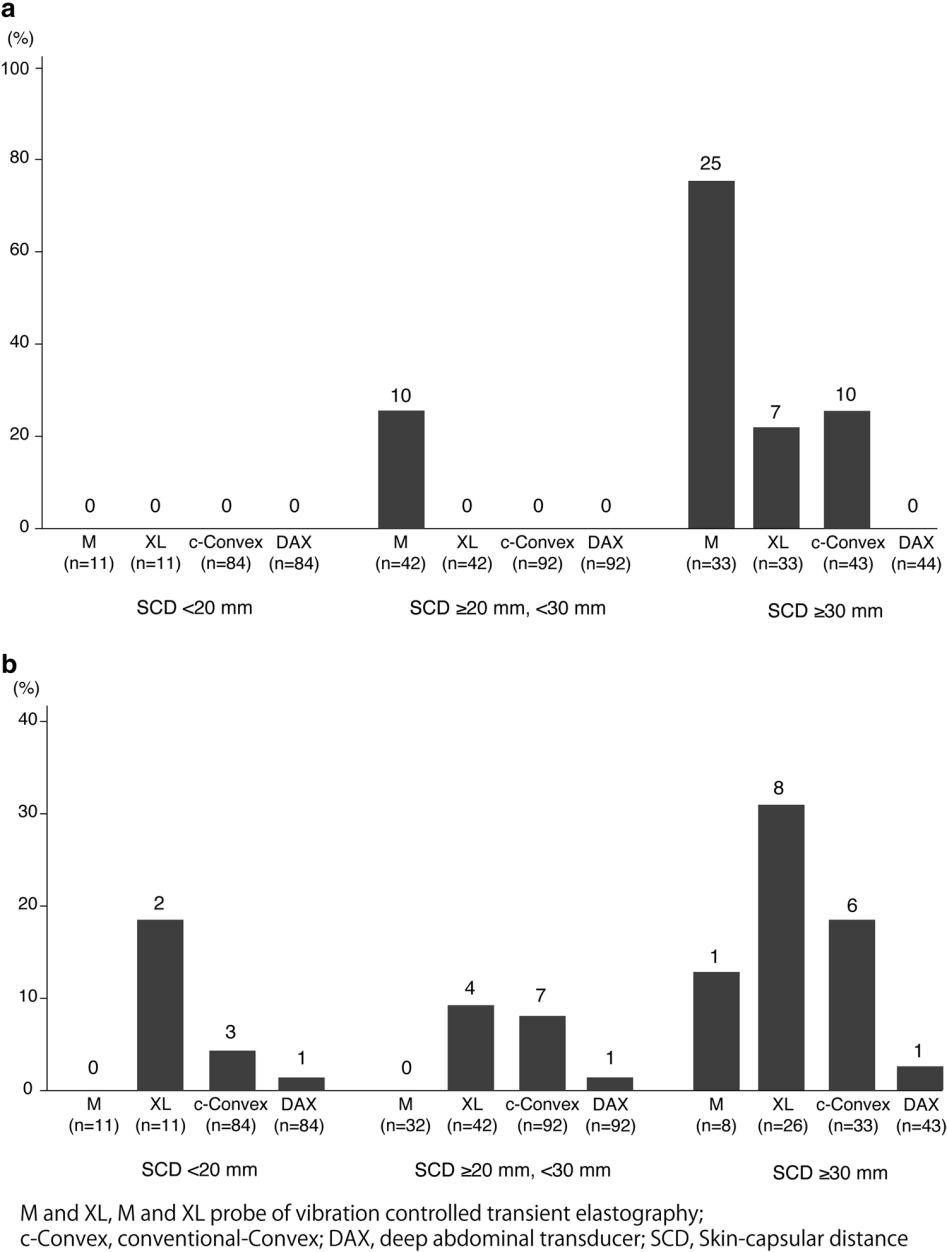
Failure of liver stiffness measurements according to SCD distance and different probes. **a** < 10 valid measurements according to different probe and SCD category. **b** Interquartile range/median > 30% according to different probe and SCD category. M and XL, M and XL probes of vibration-controlled transient elastography; *c-convex* conventional convex, *DAX* deep attenuation transducer, *SCD* skin-to-liver capsular distance

Figure 5 shows whether 10 valid LSMs or IQR/median < 0.3 were obtained. If the SCD was < 30 mm, all patients had 10 valid LSMs acquired by B-mode ultrasound elastography, while 10 patients could not undergo measurement with the M probe in this category. LSM could be successfully measured for all patients by DAX through all degrees of SCD. Especially in the patients with an SCD ≥ 30 mm, the efficacy of DAX was remarkable in the acquisition of 10 valid measurements: M probe (8/33, 24.2%), XL probe (26/33, 78.8%), c-convex probe (33/43, 76.7%), and DAX (44/44, 100%) (Fig. 5a). Figure 5b shows that inadequate measurement (IQR/median ≥ 0.3) of DAX was only observed in one patient in each degree of SCD. Moreover, in patients with SCD ≥ 30 mm, the inadequate measurement rates of the M probe (1/8, 12.5%), XL probe (8/26, 30.8%), and c-convex probe (6/33, 18.2%) were higher than those of DAX (1/43, 2.3%). The feasibility and reliability of LSM with both the c-convex probe and DAX are shown in Fig. 1. As indicated, reliable LSM using the c-convex probe was obtained in 88.1% of patients (193/219). In the remaining 11.9% (26/219) of patients, LSM using DAX could be obtained in 92.3% (24/26) of patients. Therefore, LSM with either the c-convex probe or DAX was possible in 99.1% (217/219) of patients.

**Fig. 6:**
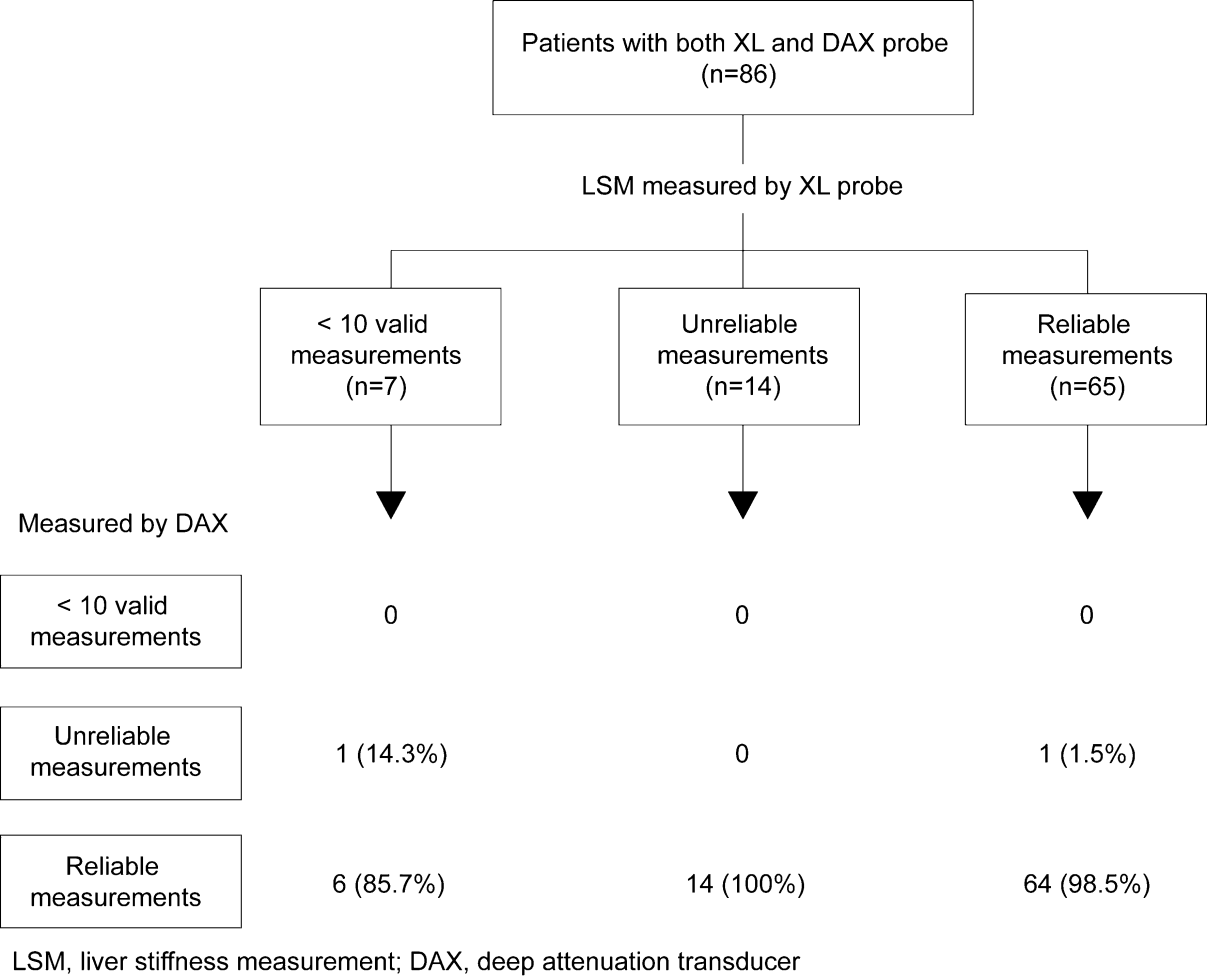
Feasibility of liver stiffness measured using DAX for patients with XL probe

A similar comparison was performed to investigate the feasibility and reliability of the XL probe and DAX by replacing the c-convex probe with the XL probe. Of the 21 patients for whom we could not acquire reliable data using the XL probe, we were unable to acquire reliable data for only one patient using DAX (Fig. 6).

### Relationship between LSM and histologic parameters

**Fig. 7:**
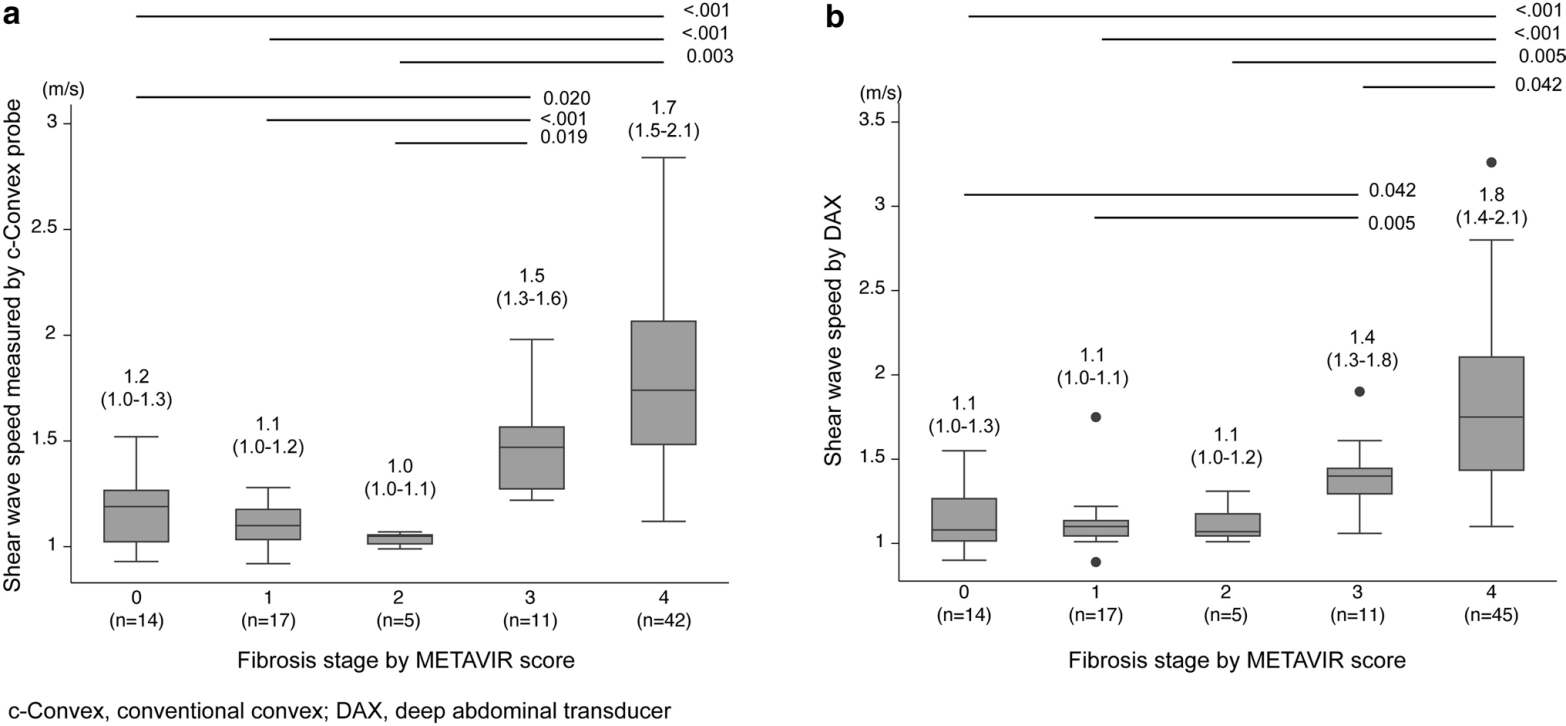
Shear wave speed measured using DAX and c-convex probes at each fibrosis stage. **a** DAX and **b** c-convex probes. *c-convex* conventional convex, *DAX* deep attenuation transducer

The median value (95% CI) of the shear wave speed (SWS) with the c-convex probe compared with the METAVIR fibrosis stage is shown in Fig. 7a. SWS was recorded as follows: F0, 1.2 m/s (1.0, 1.3); F1, 1.1 m/s (1.0, 1.2); F2, 1.0 m/s (1.0, 1.1); F3, 1.5 m/s (1.3, 1.6); and F4, 1.7 m/s (1.5, 2.1). The SWS of each METAVIR fibrosis stage differed significantly from each other (F0 vs. F3, *p* = 0.02; F1 vs. F3, p < 0.001; F2 vs. F3, *p* = 0.012; F0 vs. F4, *p* < 0.001; F1 vs. F4, *p* < 0.001; F2 vs. F4, *p* = 0.00). A similar analysis was performed using DAX for histological findings (Fig. 7b), with SWS recorded as follows: F0, 1.1 m/s (1.0, 1.3); F1, 1.1 m/s (1.0, 1.1); F2, 1.1 m/s (1.0, 1.2); F3, 1.4 m/s (1.3, 1.8); and F4, 1.8 m/s (1.4, 2.1). The SWS of each METAVIR fibrosis stage differed significantly from each other (F0 vs. F3, *p* = 0.04; F1 vs. F3, *p* = 0.01; F0 vs. F4, *p* < 0.001; F1 vs. F4, *p* < 0.001; F2 vs. F4, *p* = 0.04; F3 vs. F4, *p* = 0.04). The AUCs for diagnosis of F4 with SWS by c-convex probe and DAX were 0.916 and 0.918, respectively (*p* = 0.90, Supplemental Fig. 1). In 52 patients with non-alcoholic fatty liver disease (NAFLD), the correlations between SWS with DAX and other histological findings were also assessed. The SWS in patients with activity was higher than that in patients without activity (1.38 m/s vs. 1.01 m/s, *p* = 0.009). There was no significant difference in SWS among those with or without hepatocyte ballooning (1.42 m/s vs. 1.22 m/s, respectively; *p* = 0.272). Among each steatosis grade, there was no significant difference for SWS (S0, 1.37 m/s; S1, 1.24 m/s; S2, 1.31 m/s; S3, 1.35 m/s; S0 vs. S1, *p* = 0.975; S0 vs. S2, *p* = 0.999; S0 vs. S3, 1.000; S1 vs. S2, *p* = 0.925; S1 vs. S3, *p* = 0.831; S2 vs. S3, *p* = 0.999). In patients with only NAFLD, the AUC of the LSM for the diagnosis of F stage ≥ 3 using DAX was 0.934.

### Reliability between M and XL probes

The correlation between the M and XL probes was good (*r* = 0.865 [95% CI 0.773–0.922]) in LSM (Supplemental Fig. 2a). Different-probe reliability also revealed an excellent ICC of 0.829 (95% CI 0.639–0.912). However, Bland–Altman plots revealed that there was a statistically significant bias of 0.183 (95% CI − 0.470 to 0.836) and *r* = 0.523 (95% CI 0.346–0.664). There were significant fixed (*p* < 0.001) and proportional (*p* = 0.19) errors (Supplemental Fig. 2b).

## Discussion

The deep attenuation transducer probe was initially developed to perform abdominal ultrasound examinations for patients with severe obesity. To the best of our knowledge, there are no reports regarding deep attenuation transducers. In any degree of skin-to-liver capsular distance, liver stiffness measured with a deep attenuation transducer was more reliable than other probes. Reliable measurements were possible for obese patients who could not undergo measurements with the XL probe.

The advanced multi-D beam formation of the DAX enables the acquisition of thinner slices, enabling imaging throughout a range of up to 40 cm. This is made possible by employing an advanced form of multi-D beam formation that controls the beam thickness and formation in transmission and receive phases. NAFLD is usually observed in people who are overweight or obese. In a study of more than 8 million people, obesity was present in 51% of patients with non-alcoholic fatty liver disease and 82% of patients with non-alcoholic steatohepatitis [22]. Thus, it is important for ultrasound machines to allow for reliable examinations to be performed for obese patients.

The thickness of DAX is 28 mm, while that of the c-convex probe (such as 5C-1 probe) is 17 mm, making it more difficult to hold than the c-convex probe, which has a more refined shape. Moreover, liver stiffness is measured through the right intercostal space. There was concern that the DAX thickness would exceed the space between the ribs, thus preventing appropriate examination; this was overcome by adopting matrix arrays that allow for examination of defects with various orientations. Although inter- or intra-operator reliability was also a concern, this was excellent in our study. Despite the difficulty of holding the DAX by the operator, reproducibility was maintained. This may be why the B-mode image of the measurement site was reliably shown, unlike with VCTE probes.

When measuring liver stiffness in obese patients, those with thick subcutaneous fat and a long distance to the hepatic capsule have lower success and adequacy rates [23]. An XL probe—which contains a lower frequency and a more sensitive ultrasonic transducer, a deeper focal length, a larger vibration amplitude, and a higher depth of measurements below the skin surface—has been used in VCTE for obese patients [24]. However, a challenge remains for the XL probe: the median LSM is significantly lower than that measured with an M probe [25], possibly because non-hepatic tissue is involved in the measurement with the M probe in patients with an SCD > 25 mm. Therefore, the existing cut-offs defined in using the M probe cannot be used for the interpretation of LSMs using the XL probe [26]. Moreover, contrary to some reports [23, 27], absolute criteria to distinguish between M and XL probes in SCD thickness are unclear. Although the latest VCTE device can automatically determine probe selection, there are insufficient reports regarding this function. Thus, even though the tests were performed with the same VCTE equipment, XL and M probes need to be regarded as different tests. In our study, although the ICC was good between the M and XL probes, a fixed error was significant in the Bland–Altman analysis. If reliable LSM values are acquired using M and XL probes in patients with mild obesity, it remains difficult to determine which value should be adopted. Because the correlation coefficient between the c-convex probe and DAX was approximating 1.0, the LSM values of the c-convex probe and DAX are similar in absolute value. Regarding DAX, these issues can be identified as our results showed that it is compatible with the c-convex probe, and the results can be applied without conversion formulas. Measurements obtained with DAX can even be applied to patients without obesity. Thus, DAX could be used for all patients if one is available. However, it may be better to use a c-convex probe in patients with emaciation; insufficient intercostal muscle mass and thin subcutaneous fat may result in inappropriate measurements with DAX.

In terms of divergence of the LSM between the c-convex probe and DAX, only SCD was a significant factor according to both univariate and multivariate analyses. In patients with high SCD, variations in LSM occurred with the c-convex probe. With DAX, liver stiffness can be measured without changing probes every time, whether the person is obese or not. LSM measured by DAX not only had a high rate of reliable values but also reliable tissue findings. If liver stiffness cannot be measured with an XL probe, most patients can undergo measurements for reliable LSM by changing from an XL probe to DAX.

LSM measured by DAX was influenced by activity, while hepatocyte ballooning and steatosis did not influence it. Because the viscosity is thought to be increased by the increase in activity, the SWS was significantly influenced by the high-activity grade.

There were some limitations in this study. First, this was a retrospective cross-sectional study conducted at a single center. A prospective study should be conducted in multiple centers. Second, not all ultrasound devices are equipped with DAX; hence, versatility may be low. Third, many patients with F4 were enrolled, and other F-stage patients should be added and analyzed in the future. Fourth, 2D-SWE is currently the mainstream of LSM on ultrasound elastography, which can measure a relatively larger region of the liver than p-SWE. Presently, DAX does not support 2D-SWE. DAX has a longer measurement time than other probes, even when 1D-SWE is used. As equipment performance improves, DAX will be able to accommodate 2D-SWE in the future. Finally, a comparison of MRE and DAX in obese patients would be very interesting; a comparative study between MRE and DAX would be warranted in the future.

## Conclusion

Liver stiffness measured with a deep attenuation transducer is feasible and reliable for all patient populations. The deep attenuation transducer can provide reliable data for patients who cannot undergo measurement with the XL probe, which is now widely utilized for obese patients.

### Supplementary Information

Below is the link to the electronic supplementary material.

Supplemental Figure 1: Comparison of diagnostic ability for cirrhosis between deep attenuation transducer and conventional-convex probe

Supplemental Figure 2: Reliability between liver stiffness measurements performed using different probes. a) Scatter plots between liver stiffness measurements performed using XL and M probes. b) Bland–Altman plots show fixed bias. SWS, shear wave speed; CI, confidence interval

## Data Availability

The data sets generated and/or analyzed in this study are available from the corresponding author upon reasonable request.
