# Correction to: Deep attenuation transducer to measure liver stiffness in obese patients with liver disease

**DOI:** 10.1007/s10396-024-01423-1

**Published:** 2024-03-05

**Authors:** Masashi Hirooka, Yohei Koizumi, Yoshiko Nakamura, Ryo Yano, Kana Hirooka, Makoto Morita, Yusuke Imai, Yoshio Tokumoto, Masanori Abe, Yoichi Hiasa

**Affiliations:** 1 Department of Gastroenterology and Metabology, Ehime University Graduate School of Medicine, Shitsukawa 454, Toon, Ehime 791-0295 Japan; 2 Department of Gastroenterology, National Hospital Organization Ehime Medical Center, Yokogawara 366, Toon, Ehime 791-0281, Japan

**Correction to: Journal of Medical Ultrasonics (2023) 50:63–72** 10.1007/s10396-022-01270-y

The article “Deep attenuation transducer to measure liver stiffness in obese patients with liver disease”, written by, Masashi Hirooka, Yohei Koizumi, Yoshiko Nakamura, Ryo Yano, Kana Hirooka, Makoto Morita, Yusuke Imai, Yoshio Tokumoto, Masanori Abe and Yoichi Hiasa was originally published Online First without Open Access. After publication in volume 50, issue 1, page 63–72 the author decided to opt for Open Choice and to make the article an Open Access publication. Therefore, the copyright of the article has been changed to © The Author(s) 2022 and the article is forthwith distributed under the terms of the Creative Commons Attribution 4.0 International License, which permits use, sharing, adaptation, distribution and reproduction in any medium or format, as long as you give appropriate credit to the original author(s) and the source, provide a link to the Creative Commons licence, and indicate if changes were made. The images or other third party material in this article are included in the article’s Creative Commons licence, unless indicated otherwise in a credit line to the material. If material is not included in the article’s Creative Commons licence and your intended use is not permitted by statutory regulation or exceeds the permitted use, you will need to obtain permission directly from the copyright holder. To view a copy of this licence, visit http://creativecommons.org/licenses/by/4.0.

The original article has been corrected.

