# Supplementary figures and images for: Deep attenuation transducer to measure liver stiffness in obese patients with liver disease
Source: J Med Ultrason (2001). 2022 Dec 16;50(1):63–72. doi: 10.1007/s10396-022-01270-y (PMC10899308; doi:10.1007/s10396-022-01270-y)

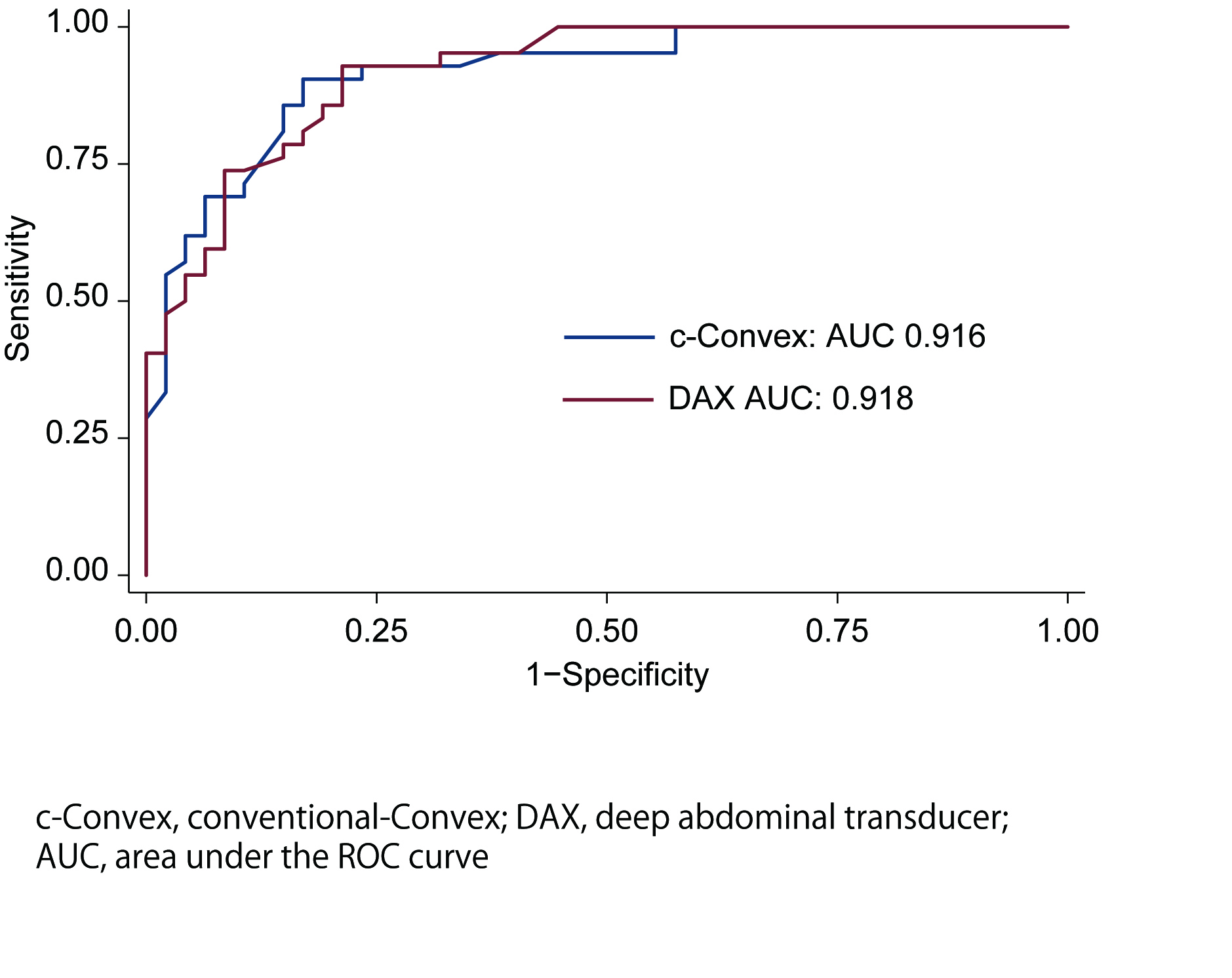

Supplement: Supplementary file 1 — Supplemental Figure 1: Comparison of diagnostic ability for cirrhosis between deep attenuation transducer and conventional-convex probe [file 10396_2022_1270_MOESM1_ESM.jpg]

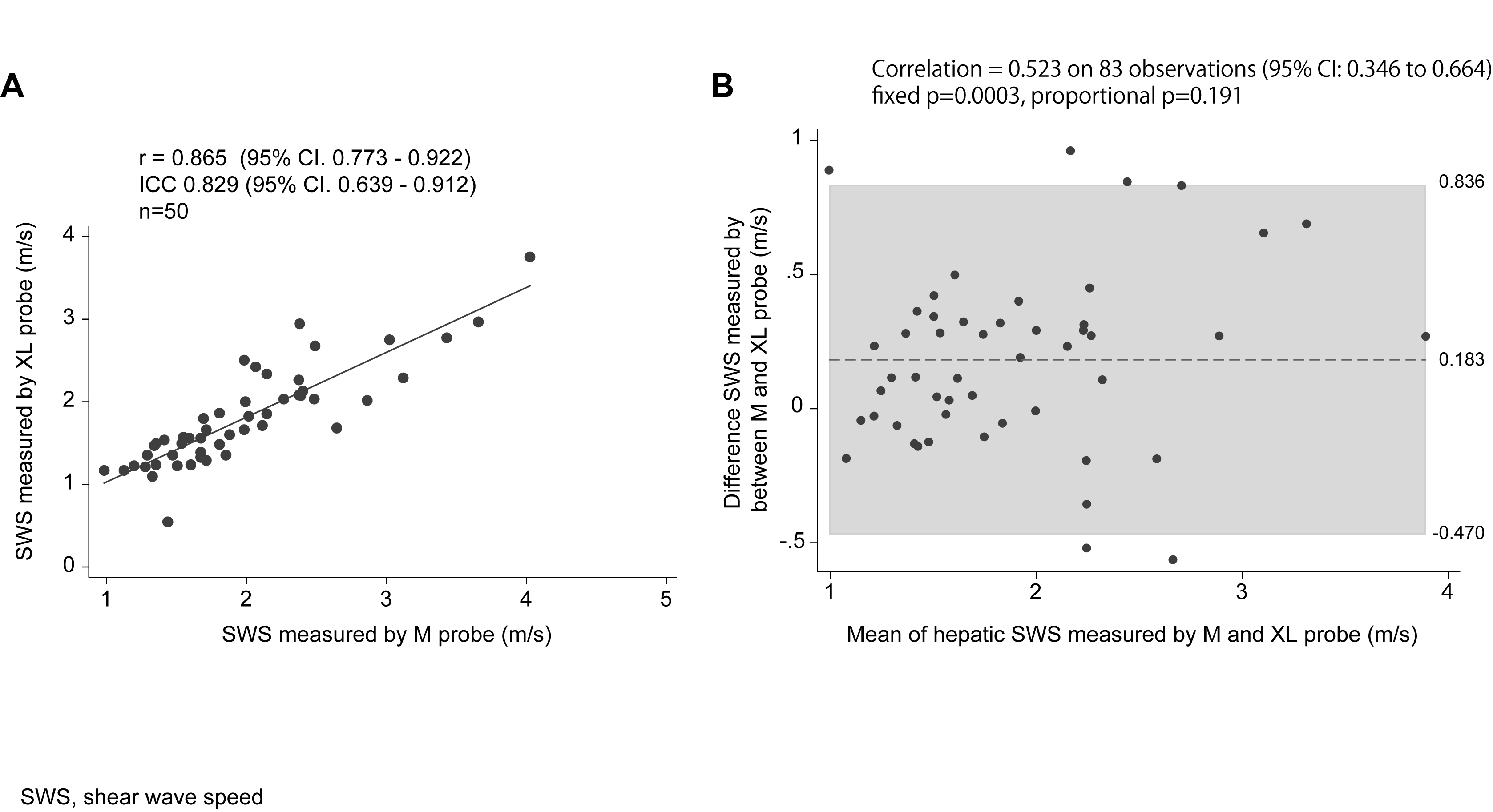

Supplement: Supplementary file 2 — Supplemental Figure 2: Reliability between liver stiffness measurements performed using different probes. a) Scatter plots between liver stiffness measurements performed using XL and M probes. b) Bland–Altman plots show fixed bias. SWS, shear wave speed; CI, confidence interval [file 10396_2022_1270_MOESM2_ESM.jpg]
